# Effect of electroacupuncture on global cerebral ischemia‑reperfusion injury in rats: A urine proteome analysis

**DOI:** 10.1002/brb3.3382

**Published:** 2024-01-08

**Authors:** Xiao Zhang, Yuting Dai, Fuguo Ma, Yuan Ma, Jiajia Wang, Xiaoxia Li, Weiwei Qin

**Affiliations:** ^1^ Department of Anesthesiology Qingdao Hospital University of Health and Rehabilitation Sciences (Qingdao Municipal Hospital) Qingdao China; ^2^ Department of Genetics and Cell Biology, Basic Medical College Qingdao University Qingdao China

**Keywords:** cerebral ischemia‑reperfusion injury, data‐independent acquisition, electroacupuncture, proteome

## Abstract

**Background:**

This study aimed to investigate dynamic urinary proteome changes of electroacupuncture (EP) on cerebral ischemia‑reperfusion (CI/R) injured rats and to explore the therapeutic biological mechanisms of EP.

**Methods:**

First, changed urinary proteins were found in EP stimulation in healthy rats. Then, we used a CI/R injury rat model induced by Pulsinelli's four‐vessel occlusion (4‐VO) method to explore the function of EP on urinary proteome in CI/R injury. Urine samples were collected for proteome analysis by liquid chromatography‐tandem mass spectrometry (LC‐MS/MS) and bioinformatics analysis.

**Results:**

In total, 384 proteins were identified, among which 47 proteins (23 upregulated, 24 downregulated) were differentially expressed with 0.6‐log FC and *p* < .05. Gene ontology analysis revealed that the cell redox homeostasis, acute‐phase response, response to lipopolysaccharide, and cellular response to glucocorticoid stimulus were significantly enriched. The partially biologically connected differential proteins were found by the Kyoto Encyclopedia of Genes and Genomes (KEGG) analysis in the EP group. With the CI/R rat model, 80 proteins (27 upregulated, 53 downregulated) were significantly changed in the CI/R rats compared to the controls. Among these differentially expressed proteins (DEPs), 23 proteins (17 upregulated, six downregulated) showed significant changes after EP treatment (0.6‐log FC change, *p* < .05). The main related biological processes were aging, immune response, acute‐phase response, liver regeneration, protein catabolic process, and response to oxidative stress. Many metabolic pathways were enriched by KEGG analysis.

**Conclusion:**

Our results indicate that the EP could alleviate cerebral damage induced by ischemia‑reperfusion through an anti‐inflammatory and metabolism regulation mechanism. The urinary proteome might reflect the pathophysiological changes in EP pretreatment in the treatment and prevention of CI/R injury.

## Graphical abstract

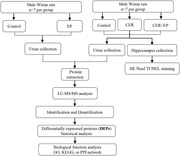

## 1 INTRODUCTION

Worldwide, one of the most important causes of mortality and disability is cerebral ischemic stroke, which accounts for about 85–87% of all stroke cases in the USA (Benjamin et al., 2019). It also causes healthcare systems a financial burden. Ischemic stroke is characterized by the permanent infarction of brain tissues, resulting in irreversible impairment of neurologic function. Until now, effective treatment for ischemic stroke can be thrombolysis and interventional therapy (Lapchak, 2015). The above treatments are a double‐edged sword. A large body of evidence demonstrates that reperfusion of ischemic brain tissues could trigger additional tissue injury, named cerebral ischemia/reperfusion (CI/R) (Nighoghossian et al., 2016; Rabinstein, 2020; Smith et al., 2019). The pathological mechanisms of CI/R are complicated and largely unclear, which include disordered metabolism, inflammatory response, and immunomodulatory pathway. Therefore, alternative approaches for CI/R are necessary to improve neurological function through various targets (George & Steinberg, 2015).

Even at an early stage, urine can reveal pathophysiological changes in the brain (An & Gao, 2015). Our previous studies revealed the predictive value of urine proteome for a sensitive reflection of CI/R pathophysiology at an early stage by using the data‐independent acquisition (DIA) proteomics technique in a rat model, which provides an ample reserve of therapeutic targets and/or biomarker candidates for subsequent studies (Sun et al., 2022). A number of proteomic analyses have assessed cerebrospinal fluid or serum, which may be more relevant to brain pathology, but these samples are much more invasive and relatively difficult to obtain. Urine, as an attractive resource for biomarker research, can be collected noninvasively and continuously.

The electroacupuncture (EP) treatment has been used for stroke rehabilitation in China for thousands of years (Lin et al., 2015). According to traditional Chinese medicine, Shenting (DU24) and Baihui (DU20), one of the branches of the Du Meridian that runs over the head, are both associated with cognitive function. Previous studies showed that EP on DU24 and DU20 is considered an effective nonpharmacologic intervention to prevent or to slow the progression of cerebral injury, which may be related to altered apoptosis and activated exosome‐carried miRNAs (Xu et al., 2022; Yip et al., 2012). Further investigation is required to elucidate the molecular processes associated with EP treatment. While previous studies have examined urine protein expressions following CI/R injury, there remains a need to explore the differential proteome profiles and the interconnected network of neuroprotective mechanisms at the proteome level in relation to EP treatment.

To understand the underlying mechanisms of cerebral injury induced by ischemia/reperfusion (I/R) and the critical roles of EP contributing to cerebral injury therapy, we employed the data mining of urine proteomics analysis among healthy male rats. The experiment was conducted in two parts. In the first part, we found the differentially expressed proteins (DEPs) after EP on DU24 and DU20 in normal rats using the DIA approach. In the second part, the same approach was used to profile the urine proteome of CI/R rats with/without EP treatment.

## 2 MATERIALS AND METHODS

### 2.1 Animals and ethics statement

Specific pathogen‐free male Wistar rats (weight 250−300 g) were provided by Jinan Pengyue Laboratory Animal Technology Co., Ltd (Jinan, China). The Medical Animal Ethics Committee of Qingdao Municipal Hospital, College of Qingdao University, Qingdao (approved ID: 2021–118) approved this experiment. All rats were housed under pathogen‐free conditions (temperature 22 ± 1˚C, humidity 50%, 12 h light/dark cycle).

First, 14 rats were acclimated for 1 week and then divided into two groups randomly (*n* = 7): control and EP groups. Then, another 21 rats were divided randomly into three groups (*n* = 7): the control group, the CI/R group, and the CI/R+EP group.

### 2.2 Establishment of the CI/R model

The rat model of transient global CI/R is prepared by Pulsinelli's four‐vessel occlusion (4‐VO) method according to the previous study (Pulsinelli & Buchan, 1988). Rats were fasted for 24 h before the operation was conducted. During the foundation of the CI/R model, rats were intraperitoneally anesthetized with 400 mg/kg chloral hydrate (Sigma, Taufkirchen, Germany). The bilateral pterygoid foramen, the bilateral vertebral arteries, and the bilateral common carotid arteries were then carefully isolated. Always, at first, the bilateral vertebral arteries were permanently occluded by electrocoagulation using a hot probe (SurgiStatTM II; Covidien, Boulder, CO, USA). Second, an 18–22 mm of monofilament nylon thread (Beijing Cinontech Co. Ltd., Beijing, China) with a rounded tip was inserted into the bilateral common carotid arteries to completely interrupt the blood flow the next day. Rats with loss of righting reflex, dilated pupils, and no response to light within 30 s were selected for later experiments. After 15 min of occlusion, the nylon thread was withdrawn to allow artery area reperfusion. Reperfusion was initiated if the thread was released to restore blood supply for 24 h. Apart from ligations and occlusions, the operation in the control group was performed essentially as above. After reperfusion, the rats were finally euthanized by decapitation, and hippocampus and urine were collected.

### 2.3 EP treatment

Rats in the CI/R+EP group received EP after the establishment of the transient global CI/R model. Rats in the EP group underwent EP treatment directly (Lin et al., 2015). The EP treatment was repeated for 30 min once daily for seven consecutive days. The EP was performed on CI/R rats at the acupoints of DU24 and DU20. The depth of the needle (0.30 mm in diameter; Dong Bang, Gyeonggi‐do, Korea) insertion was 2–3 mm, and the needle was at DU24 and DU20. The stimulation was delivered using a nerve stimulator (model SDZ‐V; Suzhou Hua Tuo Medical Instrument Co., Ltd., Suzhou, China) with a stimulating current ranging between 1 and 3 mA. The rats were sacrificed immediately after the treatment of EP.

### 2.4 Histopathology analysis

To investigate brain changes after CI/R injury, the hippocampus was harvested after the animals were sacrificed. Then, the hippocampus was perfused with phosphate‐buffered saline and embedded with 4% paraformaldehyde overnight at 4°C. Intact hippocampus tissue was isolated and subsequently subjected to fixation, washing, dehydration, and processing for transparency, as well as preparation of paraffin‐embedded blocks. The tissue was sectioned (5 μm), and then deparaffinized using xylene and ethanol, transitioning to water, and subsequently stained with 0.5% hematoxylin and 0.5% eosin (Beyotime, Shanghai, China). The pathological morphology of hippocampal tissues was observed under a microscope 200× (Olympus, Tokyo, Japan). Besides, Nissl staining was used to reveal the histopathological lesions.

### 2.5 TUNEL staining

The hippocampus tissue, which was embedded in paraffin, underwent fixation in xylene, hydration using a gradient of ethanol, and permeabilization with proteinase K for a duration of 30 min. Subsequently, the hippocampus tissue was exposed to TUNEL reagent for 60 min at a temperature of 37°C, followed by photography. The cells labeled in brown were identified as TUNEL‐positive cells.

### 2.6 Urine sample collection and preparation

Urine samples were collected using metabolic cages for 4 h. After centrifugation at 2000×*g* for 30 min, urinary proteins were extracted by ethanol precipitation. The urinary proteins were digested by 10‐kDa filter‐aided trypsin as described before. Briefly, equal amounts (100 μg) of protein were loaded onto a 10‐kDa filter unit (Pall, Port Washington, NY, USA). The proteins were reduced using a solution of 4.5 mM DTT for 1 h at 37°C; then with an alkylation solution of 10 mM iodoacetamide at room temperature in the dark. The peptides, digested with trypsin 14 h at 37°C, were desalted using Oasis HLB cartridges (Waters, Milford, MA, USA) before liquid chromatography‐tandem mass spectrometry (LC‐MS/MS) analysis. With the help of a high‐pH reversed‐phase peptide fractionation kit supplied by Thermo Pierce (Waltham, MA, USA), the pooled peptide samples were mixed and fractionated. Using Spin Columns (Thermo Pierce, USA), 60 μg of the pooled peptide sample was centrifuged to collect beads. The lyophilized peptides were prepared for LC‐MS/MS analysis (Qin et al., 2021).

### 2.7 The LC‐MS/MS analysis

The DDA‐MS and DIA‐MS data were acquired by an Easy nLC 1000 HPLC system coupled with an Orbitrap Fusion Lumos Tribrid mass spectrometer (Thermo Scientific, USA). For calibration, samples were spiked with the calibration kit (iRT, Biognosys, Schlieren, Switzerland) at a ratio of 1:20 (v/v). The digested peptides were trapped on the C18 column (3 μm, 75 μm × 2 cm, 100 A). The eluted gradient was 5–30% for 90 min (buffer: 80% acetonitrile 0.1% in formic acid; flow rate of 0.8 μL/min). Parameters were set as follows: the full scan was set at a resolution of 60,000 over an *m*/*z* range of 350–1200, followed by DIA scans with a resolution of 30,000, high‐energy collision dissociation (HCD) collision energy of 32%, automatic gain control (AGC) target of 1E6, and maximal injection time of 50 ms.

### 2.8 The DIA quantification analysis

The Sequest HT search algorithm combined with Proteome Discoverer (Thermo Scientific, San Jose, CA, USA, version 2.1) was used to search MS/MS spectra to identify proteins. Search parameters were set as follows: MS tolerance of 10 ppm, an MS2 tolerance of 0.02 Da, and two missed cleavages allowed. All other parameters retained the default settings. The DIA‐MS raw files were imported to Spectronaut Pulsar with the default settings. In brief, all results were filtered by an false discovery rate (FDR) of 1%. Peptide intensity was calculated by summing the peak areas of their respective fragment ions for MS2.

### 2.9 Biological function analysis

Proteomic analysis was used to detect differential proteins in this study after EA. The DEP_S_ in the urine of rats were further analyzed after CI/R. Gene ontology (GO) analysis was applied for urinary protein function analysis in the first experiment (http://www.geneontology.org/). To construct significantly enriched functional pathways, the Kyoto Encyclopedia of Genes and Genomes (KEGG) was used (https://string‐db.org/). The protein–protein interaction analysis was obtained within the analysis by STRING database (https://string‐db.org). Statistical analysis of the experimental data was performed using the “Wu Kong” platform (https://www.omicsolution.org/wkomics/main/). Differences between groups were tested by a two‐sample independent *t‐*test or one‐way analysis of variance. Significance was set at a *p*‐value of < 0.05 and a log FC of 0.6.

## 3 RESULTS

### 3.1 Urinary proteome changes in EP stimulation in the rats

A rat model of EP was established to explore the urinary proteome changes in EP treatment as previously described (Lin et al., 2015). In the first experiment, 14 urine samples were analyzed by LC‐MS/MS, seven of which were from the EP rats and seven from the control rats. Overall, 384 high‐confidence urinary proteins were identified (FDR < 1%), among which 47 proteins (23 upregulated, 24 downregulated) were differentially expressed (log FC absolute value > 0.6, *p* < .05; Table 1). The top 20 ranked with log FC is marked in red.

**TABLE 1:** Details of the differentially expressed urinary proteins in EP rats.

Uniprot ID	Protein names	*p*‐value	log FC	Trend
P08932	T‐kininogen 2	.002	2.418	Up
P02761	Major urinary protein	.003	−1.928	Down
P46413	Glutathione synthetase	.020	−1.818	Down
P01048	T‐kininogen 1	.000	1.795	Up
P02764	Alpha‐1‐acid glycoprotein	.001	1.545	Up
Q64602	Kynurenine/alpha‐aminoadipate aminotransferase, mitochondrial	.027	−1.450	Down
Q9QY16	ATP‐dependent RNA helicase DDX25	.049	−1.396	Down
P19468	Glutamate–cysteine ligase catalytic subunit	.015	−1.384	Down
Q6Q0N1	Cytosolic nonspecific dipeptidase	.012	−1.305	Down
Q63530	Phosphotriesterase‐related protein	.006	−1.269	Down
P27645	Low‐affinity immunoglobulin gamma Fc region receptor III	.000	1.187	Up
P15473	Insulin‐like growth factor‐binding protein 3	.032	1.178	Up
P07150	Annexin A1	.023	−1.154	Down
Q9Z0V6	Thioredoxin‐dependent peroxide reductase, mitochondrial	.021	−1.058	Down
Q63556	Serine protease inhibitor A3M	.007	1.053	Up
Q6P6S9	Ectonucleoside triphosphate diphosphohydrolase 5	.035	−1.046	Down
P10247	H‐2 class II histocompatibility antigen gamma chain	.003	1.043	Up
P10959	Carboxylesterase 1C	.029	−1.014	Down
Q6P9T8	Tubulin beta‐4B chain	.023	−0.942	Down
Q64573	Liver carboxylesterase 4	.021	−0.863	Down
P60711	Actin, cytoplasmic 1	.009	−0.858	Down
O70534	Protein delta homolog 1	.006	−0.853	Down
P70619	Glutathione reductase	.024	−0.851	Down
P47967	Galectin‐5	.036	0.849	Up
Q62740	Secreted phosphoprotein 24	.047	0.843	Up
P50116	Protein S100‐A9	.001	−0.839	Down
P81828	Urinary protein 2	.009	0.828	Up
P19112	Fructose‐1,6‐bisphosphatase 1	.028	−0.815	Down
Q5FVR0	T‐cell immunoglobulin and mucin domain‐containing protein 2	.013	0.801	Up
P23680	Serum amyloid P‐component	.002	0.786	Up
P07154	Cathepsin L1	.027	0.767	Up
P52759	2‐iminobutanoate/2‐iminopropanoate deaminase	.023	−0.758	Down
P0CG51	Polyubiquitin‐B	.030	−0.758	Down
P70490	Lactadherin	.032	0.753	Up
P15083	Polymeric immunoglobulin receptor	.014	0.739	Up
P04218	OX‐2 membrane glycoprotein	.012	0.713	Up
Q80WD1	Reticulon‐4 receptor‐like 2	.010	0.711	Up
P09006	Serine protease inhibitor A3N	.018	0.705	Up
P68370	Tubulin alpha‐1A chain	.017	−0.694	Down
P63018	Heat shock cognate 71 kDa protein	.050	−0.671	Down
P19804	Nucleoside diphosphate kinase B	.034	−0.665	Down
Q6TUD4	Protein YIPF3	.025	0.661	Up
Q4V8I1	Endothelial protein C receptor	.018	0.659	Up
Q01460	Di‐N‐acetylchitobiase	.030	0.658	Up
P08460	Nidogen‐1	.003	0.632	Up
Q9WUK5	Inhibin beta C chain	.008	0.614	Up
Q9ESV6	Glyceraldehyde‐3‐phosphate dehydrogenase, testis‐specific	.038	−0.605	Down

### 3.2 GO analysis and enriched KEGG pathways of differential proteins in EP rats

**FIGURE 1:**
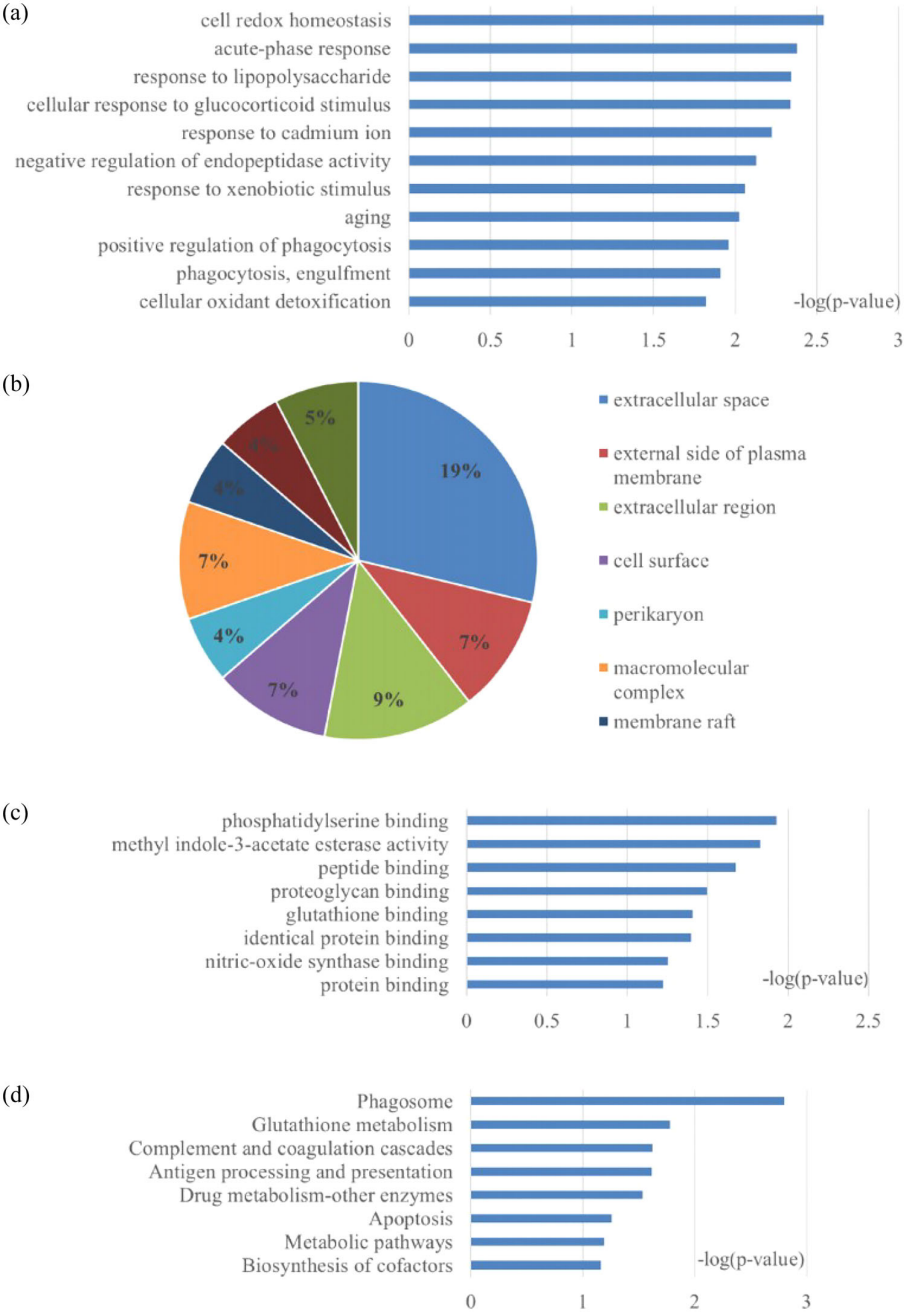
GO analysis and KEGG pathways of the differential proteins in EP rats: (a) biological process; (b) cellular component; (c) molecular function; (d) KEGG pathways.

Using the GO database, differential proteins were annotated by biological process, cell component, and molecular function to understand the function and feature of the proteins, as shown in Figure 1. Biological processes tended to be a high level of enriched cell redox homeostasis, acute‐phase response, response to lipopolysaccharide, and cellular response to glucocorticoid stimulus. Cellular component analysis indicated the DEPs were significantly enriched in the extracellular space, extracellular exosome, and extracellular region, while some were integral components of the membrane. In addition, phosphatidylserine binding, methyl indole‐3‐acetate esterase activity, and peptide binding were highly represented in the molecular function category. In order to clarify and understand the biological functions of differential proteins, we constructed the KEGG pathways by using the KEGG database. The enriched KEGG pathways were mainly involved in transport and catabolism (phagosome), metabolism (drug metabolism, biosynthesis of cofactors, cysteine, and methionine metabolism, glycolysis/gluconeogenesis, glutathione metabolism, and nucleotide metabolism), immune system (antigen processing and presentation), and cell growth and death (apoptosis), as shown in Figure 1.

### 3.3 Protein–protein interactions of DEPs associated with EP

**FIGURE 2:**
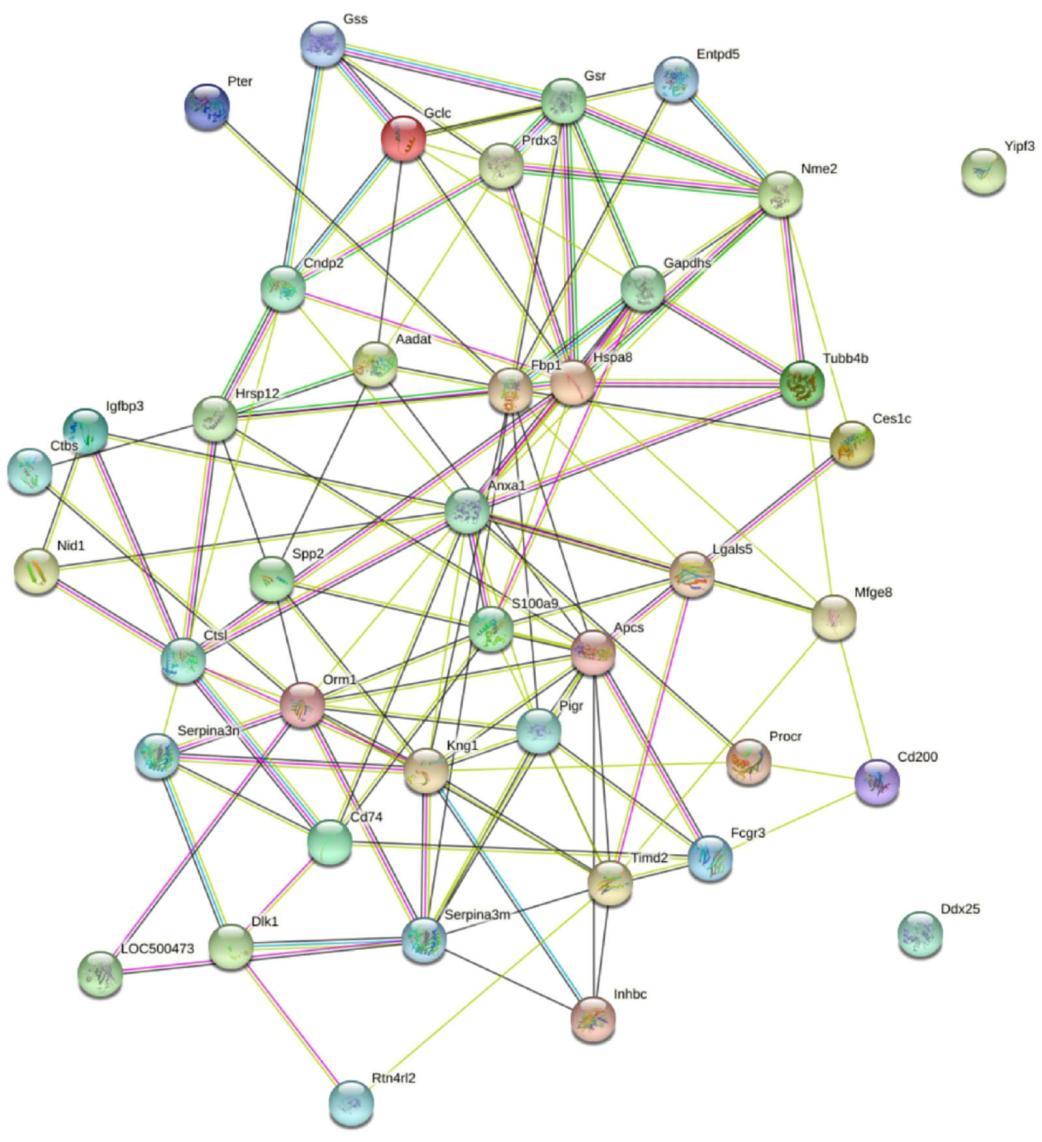
PPI network analysis of the 47 differentially changed proteins in EP rats. Network Stats: number of nodes: 41; number of edges: 127; average node degree: 6.2; average local clustering coefficient: 0.403; expected number of edges: 47; PPI enrichment *p*‐value < 1.0e‐16.

To distinguish the pathogenic mechanisms in EP, the protein–protein interaction (PPI) network was constructed using STRING. The PPI network in the STRING database demonstrated 41 nodes and 127 interactions, including an average node degree of 6.2. These proteins exhibited a higher number of interactions between themselves within the mentioned network. This can be attributed to the fact that many proteins located at the core in interaction networks, such as PRDX3 (thioredoxin‐dependent peroxide reductase, mitochondrial), GSR (glutathione reductase), TUBB4B (tubulin beta‐4B chain), HSPA8 (heat shock cognate 71 k Da protein), ANXA1 (annexin A1), S100A9 (protein S100‐A9), APCS (serum amyloid P‐component), ORM1 (alpha‐1‐acid glycoprotein), KNG1 (T‐kininogen 1), and SERPINA3M (serine protease inhibitor A3M), which are shown in Figure 2. This illustrates the fact that these proteins might play important roles in EP treatment.

### 3.4 Protective effects of EP on neurological damage after CI/R injury

**FIGURE 3:**
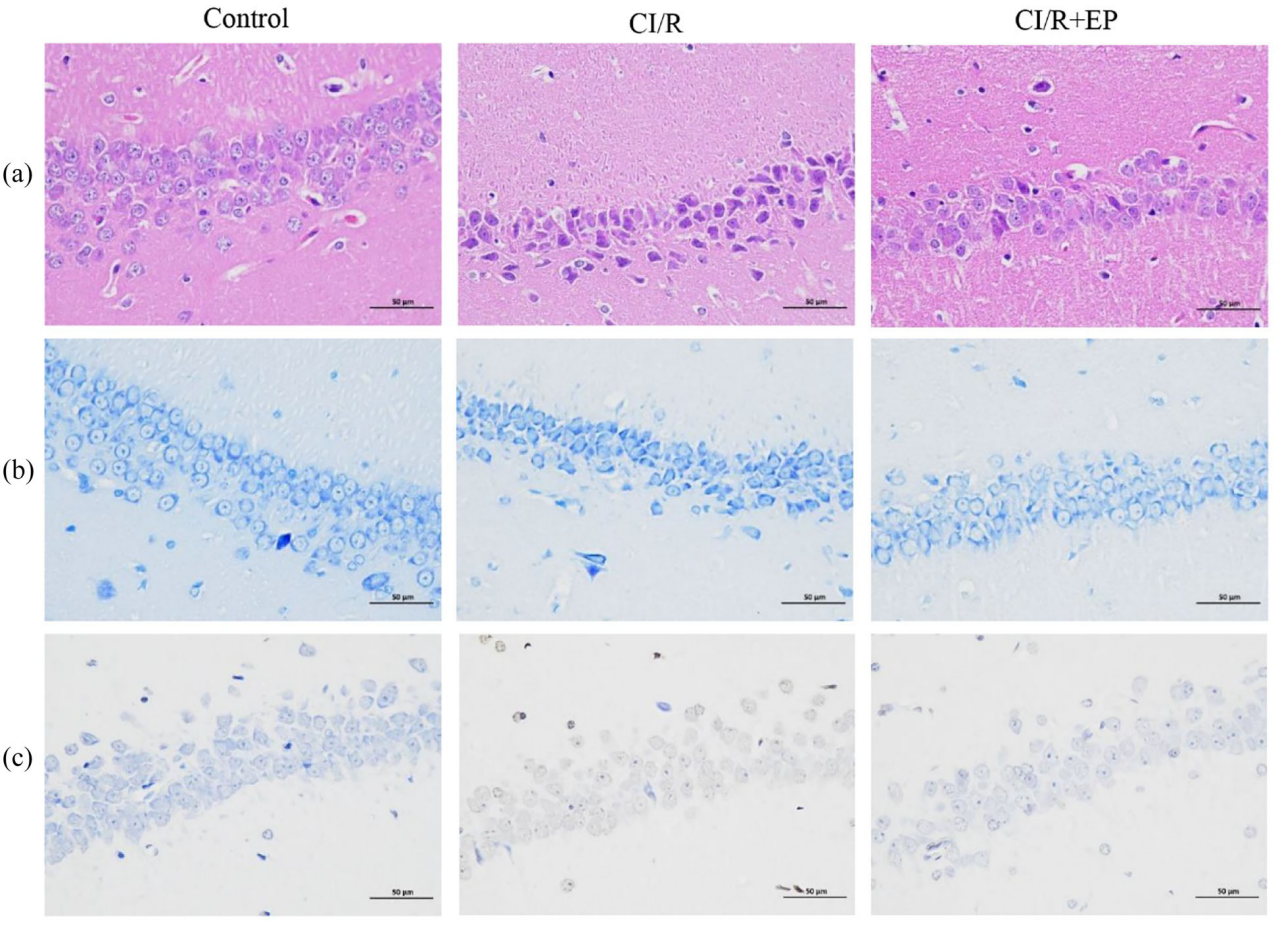
Protective effects of EP on neurological damage after CI/R injury. (a) HE staining; (b) Nissl staining; (c) TUNEL staining. Scale bars = 50 μm.

To investigate the effect of EP on CI/R in rats, we established a transient global CI/R model. The extent of hippocampal neuronal pathology in rats was assessed using HE staining and Nissl staining, while TUNEL staining was employed to detect alterations in hippocampal neuronal apoptosis. Hippocampal HE staining of the control group showed there was no obvious abnormality concerning the hippocampal region. The neurons in the hippocampus had different degrees of lesions and necrosis, with smaller volume, greater architectural destruction, deeper staining nuclei, and cytosolic coagulation in the CI/R group. These alterations were ameliorated by EP treatment. Cells in the CI/R+ EP group displayed a better morphology, and smaller necrotic cells compared with those in the CI/R group (Figure 3a). Nissl staining of the hippocampus demonstrated highly dense pyramidal layer neurons, and abundant Nissl bodies in the control‐operated rats. In contrast, I/R‐treated rats exhibited neuronal pyknosis and a significant loss of neurons. Compared to the CI/R group, seven consecutive days of EP treatment reversed the pathological changes and improved the neuronal damage of the hippocampus (Figure 3b). Furthermore, TUNEL staining revealed a significantly higher count of brown‐labeled apoptotic cells in the CI/R group compared to both the control group and the CI/R+ EP group. Remarkably, treatment with EP exhibited a protective effect against neuronal apoptosis (Figure 3c). Collectively, these findings indicate that EP treatment ameliorated the pathological damage of hippocampal neurons and mitigated apoptosis in CI/R rats.

### 3.5 The changes in urinary proteome in CI/R rats after EP treatment

**FIGURE 4:**
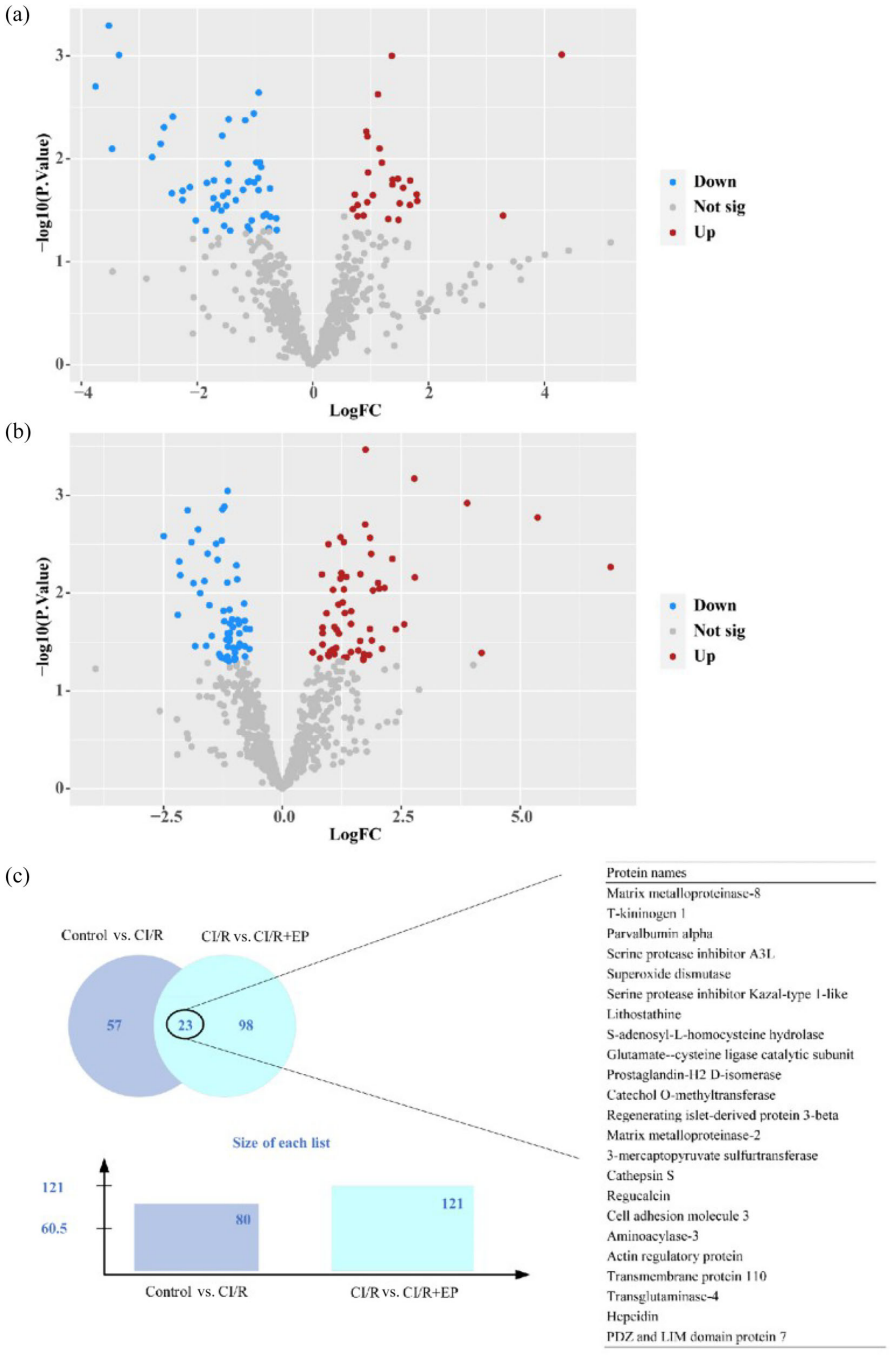
Volcano Plot and Venn diagram of the DEPs among the three groups. (a) The DEPs of the control group and CI/R group. (b) The DEPs of the CI/R group and CI/R+ EP group. (c) The intersection of differential proteins of the above two comparisons.

We analyzed a total of 21 samples of rat models in the second experiment by the LC‐DIA‐MS/MS workflow. The screening criteria for a differential protein were absolute log FC >  0.6 and *p* < 0.05. After screening, a total of 700 proteins were found for analysis. Among the 700 proteins, 80 DEPs were identified, of which 53 were downregulated and 27 were upregulated in the CI/R group compared with the control group. Similarly, 61 proteins were downregulated in the CI/R+ EP group compared with the CI/R group, and 60 proteins were upregulated. The proteomic analysis result is shown in Figure 4a,b. The intersection of differential proteins of the above two comparisons was obtained and is shown in the Venn diagram. Ultimately, 23 proteins were selected by taking the intersection. For designations, please refer to Figure 4c.

### 3.6 The DEPs of EP treatment against CI/R

A total of 23 core DEPs were obtained from the intersection of the two sets of proteins described. Table 2 shows the relative intensity and changed trend of DEPs from before to after CI/R and EP. Among them, five proteins were upregulated by CI/R and downregulated by EP; two proteins were both upregulated by CI/R and EP; 15 proteins were downregulated by CI/R and upregulated by EP; one protein was both downregulated by CI/R and EP (Table 2). Of those proteins, 11 proteins have been shown to be involved in brain disease. In addition, five proteins are shown to bind to neuroinflammation and cerebral ischemia, including parvalbumin alpha, superoxide dismutase, S‐adenosyl‐l‐homocysteine hydrolase, 3‐mercaptopyruvate sulfurtransferase, and cathepsin S (Table 2). The “top three” abundantly expressed proteins in the hippocampus of rats were parvalbumin alpha, serine protease inhibitor Kazal‐type 1‐like, and transglutaminase‐4, which were annotated with red dashes.

**TABLE 2:** The DEPs of EP treatment against CI/R.

Uniprot ID	Protein names	Control versus CI/R		CI/R versus CI/R+EP		Disease markers
		LogFC	Trend	LogFC	Trend	
O88766	Matrix metalloproteinase‐8	−1.33	Down	1.14	Up	Postmeningitidal neurological sequelae (Leppert et al., 2000)
P01048	T‐kininogen 1	0.77	Up	0.93	Up	
P02625	Parvalbumin alpha	−3.47	Down	2.31	Up	Neuroinflammation (Deng et al., 2020)
P05544	Serine protease inhibitor A3L	−1.06	Down	−1.16	Down	
P07632	Superoxide dismutase	−0.94	Down	0.79	Up	Cerebral ischemia (Mršić‐Pelčić et al., 2012; J. Wang et al., 2019)
P09656	Serine protease inhibitor Kazal‐type 1‐like	−2.42	Down	3.89	Up	
P10758	Lithostathine	−1.72	Down	1.82	Up	
P10760	S‐adenosyl‐l‐homocysteine hydrolase	1.80	Up	−1.73	Down	Hypoxic‐ischemic injury (Spinelli et al., 2021)
P19468	Glutamate–cysteine ligase catalytic subunit	1.80	Up	−2.50	Down	Neurodegenerative diseases (Fan et al., 2020)
P22057	Prostaglandin‐H2 d‐isomerase	−0.90	Down	0.83	Up	Brain tumors (Ghantasala et al., 2021)
P22734	Catechol O‐methyltransferase	−1.12	Down	1.00	Up	Alzheimer's (Perkovic et al., 2018) Schizophrenia (Aghamaleki‐Sarvestani et al., 2020)
P25031	Regenerating islet‐derived protein 3‐beta	−1.72	Down	1.71	Up	
P33436	Matrix metalloproteinase‐2	0.77	Up	0.85	Up	Glioma (Pinheiro et al., 2021; Yu‐Ju Wu et al., 2020)
P97532	3‐Mercapto pyruvate sulfurtransferase	−1.65	Down	2.04	Up	OGD/R injury (Zhang, Chen, et al., 2020)
Q02765	Cathepsin S	−1.46	Down	1.84	Up	Microglial function Neuroinflammation (L. Cao et al., 2018; Nakanishi, 2020)
Q03336	Regucalcin	0.77	UP	−1.15	Down	
Q1WIM3	Cell adhesion molecule 3	−1.71	Down	1.87	Up	
Q5M876	Aminoacylase‐3	1.50	UP	−1.31	Down	
Q6AYC4	Actin regulatory protein	−1.01	Down	1.22	Up	
Q7TSW6	Transmembrane protein 110	1.38	Up	−2.00	Down	
Q99041	Transglutaminase‐4	−3.35	Down	2.38	Up	
Q99MH3	Hepcidin	−1.83	Down	1.91	Up	Brain iron homeostasis (Vela, 2018)
Q9Z1Z9	PDZ and LIM domain protein 7	−2.25	Down	1.45	Up	

### 3.7 GO analysis and KEGG analysis of the DEPs of EP treatment against CI/R

**FIGURE 5:**
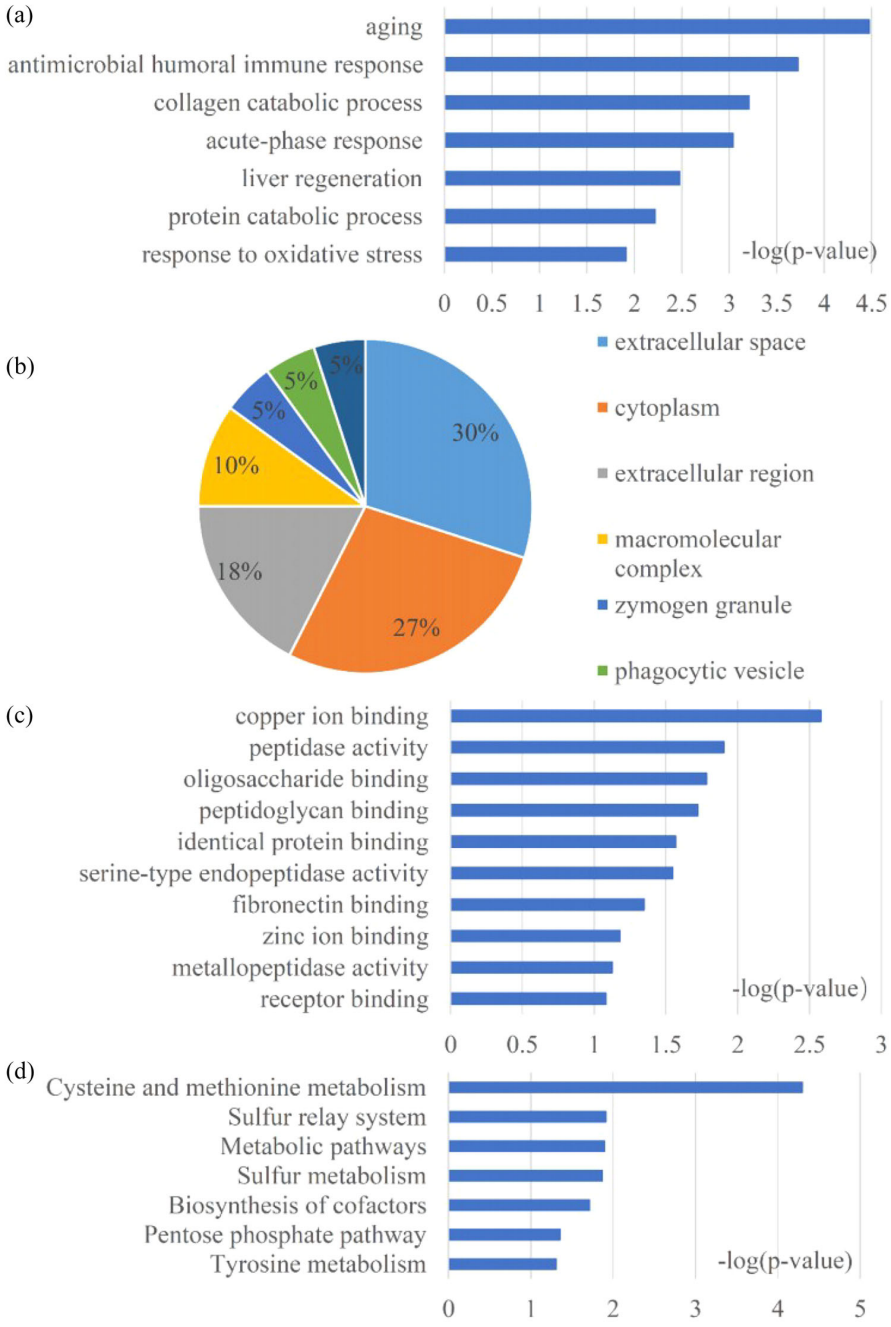
GO analysis and KEGG analysis of the DEPs of EP treatment against CI/R: (a) biological process; (b) cellular component; (c) molecular function, and (d) the canonical pathways.

To dissect the biological differences among these DEPs, we annotated the biological functions of 23 differential proteins. Three primary biological pathways were identified, which include aging, antimicrobial humoral immune response, and collagen catabolic process. GO functional analysis was used to classify the cellular components of these DEPs. Most of the proteins correspond to six cellular components, and the top three were extracellular space, cytoplasm, and extracellular region. Molecular functions mainly included copper ion binding, peptidase activity, and oligosaccharide binding. The major KEGG pathways of these differential proteins include cysteine and methionine metabolism, sulfur relay system, and metabolic pathways (Figure 5).

## 4 DISCUSSION

EP, a treatment combined with mechanical and electrical stimulation, is an integral component of Chinese traditional culture, which is making outstanding contributions to the inheritance of the Chinese nation (Bao et al., 2017). It could improve the differentiation of endogenous neurogenesis and brain function after ischemic and reperfusion (Zhang, Jin, et al., 2020). The specific mechanisms are still relatively unclear. We have elaborated on dynamic changes of urinary proteome after EP treatment in healthy rats in the first experiment. The urinary proteome identified 47 significant proteins in total, and the functional enrichment was closely related to the physiology and pathophysiology of ischemia and reperfusion brain injury. From the results mentioned above, it is indicated that the profile of urinary proteins might reflect changes in neurological functions to some extent.

Anti‐shock therapy, thrombolytic therapy, cardiopulmonary bypass, cardiac arrest, and cardiopulmonary‐cerebral resuscitation, etc. may be in part responsible for ischemia and reperfusion brain injury in clinical settings (Guan et al., 2020). Previous studies support that metabolic dysfunction (oxygen and glucose deprivation, and restoration), inflammation, and immune response are the main physiopathological mechanisms of CI/R (Jin et al., 2018; Yang et al., 2019). Our study found that the significantly changed proteins were mostly related to the immune response as well as metabolic pathways, which was similar to previous work. Furthermore, CI/R injury may lead to liver injury (Chen et al., 2018). That's also one of the reasons why we found that liver regeneration occupied an important status in functional analyses. To date, the CI/R injury is still a complex physiological and pathological process with unclear pathomechanisms, despite ample efforts that have been made (Huang et al., 2020). In addition, there is a lack of effective prognosis and diagnostic markers for patients with CI/R injury. Previous research has shown both EP and mild hypothermia prevented neuron damage induced by the ischemia‐reperfusion (Dai et al., 2015; W.‐B. Wang et al., 2016). However, the concrete mechanism was not clear. Based on the first experiment, to further explore the effect of EP on CI/R injury, we designed the second experiment with EP treatment. Urine samples were collected from seven control rats, seven CI/R rats, and seven EP rats for proteome analysis to confirm the DEPs. In total, 23 DEPs were found in the urine samples among the three groups. In addition, our findings suggested that proteins involved in the immune response, metabolism pathway, and inflammation were responsible for improving CI/R after EP treatment. Expression of 23 proteins was altered in response to CI/R injury as well as EP treatment accompanied by improved neurological conditions, suggesting that they might be utilized to represent candidate biomarkers for severity of CI/R and treatment efficacy of EP. Of those proteins, 11 proteins were identified and may be involved in brain disease, among these, five proteins (parvalbumin alpha, superoxide dismutase, S‐adenosyl‐l‐homocysteine hydrolase, 3‐mercaptopyruvate sulfurtransferase, and cathepsin S) are particularly related to neuroinflammation and cerebral ischemia. The three top‐ranked proteins of the most highly differentially expressed before and after intervention are parvalbumin alpha, serine protease inhibitor Kazal‐type 1‐like, and transglutaminase‐4. To our knowledge, parvalbumin alpha has been previously reported to be related to neuroinflammation (Deng et al., 2020), while two additional proteins have not been reported in the literature.

We found a reduction (log FC of −3.47) in parvalbumin alpha related to control and was subsequently elevated to 2.31‐log FC related to CI/R. Parvalbumin alpha, an EF‐hand Ca^2+^‐binding protein family member, was previously identified as an anatomical marker of the GABAergic neurons, and another study reported parvalbumin alpha as an antioxidant molecule associated with the brain (E. A. Permyakov & Uversky, 2022; S. E. Permyakov et al., 2014). The relationship between the clinical relevance and differences in the proteomic was uncertain since the experiment was generated in animals. In the EP‐treatment group, levels of serine protease inhibitor Kazal‐type 1‐like and transglutaminase‐4 were significantly upregulated in urine (by 3.89 and 2.38‐log FC respectively). Serine protease inhibitor Kazal‐type 1‐like appears highly expressed in the gastrointestinal tract, pancreas, urological, and lymphoid tissues and could induce angiogenesis and the transendothelial migration by activating the mitogen‐activated protein kinase pathway (Luo et al., 2022). Interestingly, serine protease inhibitor Kazal‐type 1‐like is not expressed in the brain. One of the possible explanations is that it may readily penetrate the brain via a compromised blood–brain barrier. The result suggested that serine protease inhibitor Kazal‐type 1‐like may improve brain functional activity through changes in the activity of the digestive system. Furthermore, in our study, compared with the CI/R group, EP intervention led to a significant upregulation of transglutaminase‐4, which has not been studied before. Transglutaminase‐4, a member of the TGase family, is detected in male tissues, especially, prostate and skeletal muscle associated with the mammalian reproductive process (membrane micromotion, cell and matrix adhesion, the epithelial and mesenchymal transition, etc.; Z. Cao et al., 2013; Jiang & Ablin, 2011). This difference in organ‐specific expression suggests EP treatment may be sex selective, and the treatment outcomes may also differ based on sex. Thus, of course, further studies on different sexes of rats need to be done in the future. In particular, EP treatment attenuated CI/R damage accompanied by a fewer inflammatory reaction. The primary mechanism for this is the altered proteins‐associated signaling pathways and protein–protein interaction networks.

This study had some limitations. First, this is the small sample size (21 rat urine samples) of urine proteomic study, and long‐term larger human sample experiments are required to identify such biomarkers and elucidate their associated mechanisms of EP for treating CI/R. In addition, details of EP treatment planning, follow‐up treatment strategies, and prognoses need to be explored further.

In summary, we analyzed the urine proteomes of CI/R rats receiving EP treatment, finding that proteomic analysis of urine can provide insight into the therapeutic biological mechanism of EP. This could promote more individualized treatment regimens and inform different prognoses, which make treatment more targeted.

## AUTHOR CONTRIBUTIONS

**Xiao Zhang**: Writing—original draft; resources; project administration; writing—review and editing. **Yuting Dai**: Visualization; writing—review and editing; resources. **Fuguo Ma**: Visualization; methodology. **Yuan Ma**: Methodology; data curation; visualization. **Jiajia Wang**: Conceptualization; data curation; formal analysis. **Xiaoxia Li**: Investigation; data curation; software. **Weiwei Qin**: Supervision; validation; project administration; funding acquisition.

## CONFLICT OF INTERESTS STATEMENT

The authors declare that they have no competing interests.

### PEER REVIEW

The peer review history for this article is available at https://publons.com/publon/10.1002/brb3.3382.

## Data Availability

The datasets used and/or analyzed during the current study are available from the corresponding author upon reasonable request.
